# Supplementary material for: Targeting mGlyR with nanobodies for depression
Source: Nat Commun. 2026 Jan 26;17:831. doi: 10.1038/s41467-026-68339-x (PMC12834959; doi:10.1038/s41467-026-68339-x)
Supplement: Supplementary file 1 — Supplementary Information [file 41467_2026_68339_MOESM1_ESM.pdf]

SUPPLEMENTARY MATERIALS

Targeting mGlyR with nanobodies for depression

Thibaut Laboute[1], Stefano Zucca[1,#], Omar K. Sial[1,#], Mansi Sharma[2,#], Gloria Brunori[1,#], Shikha Singh[3], KV Nageswar[2], Haiyong Peng[4], Christoph Rader[4], Jérôme AJ Becker[5,6], Julie Le Merrer[5,6], Appu K. Singh[2,7,*], Kirill A. Martemyanov[1,*]

SUPPLEMENTAL FIGURES

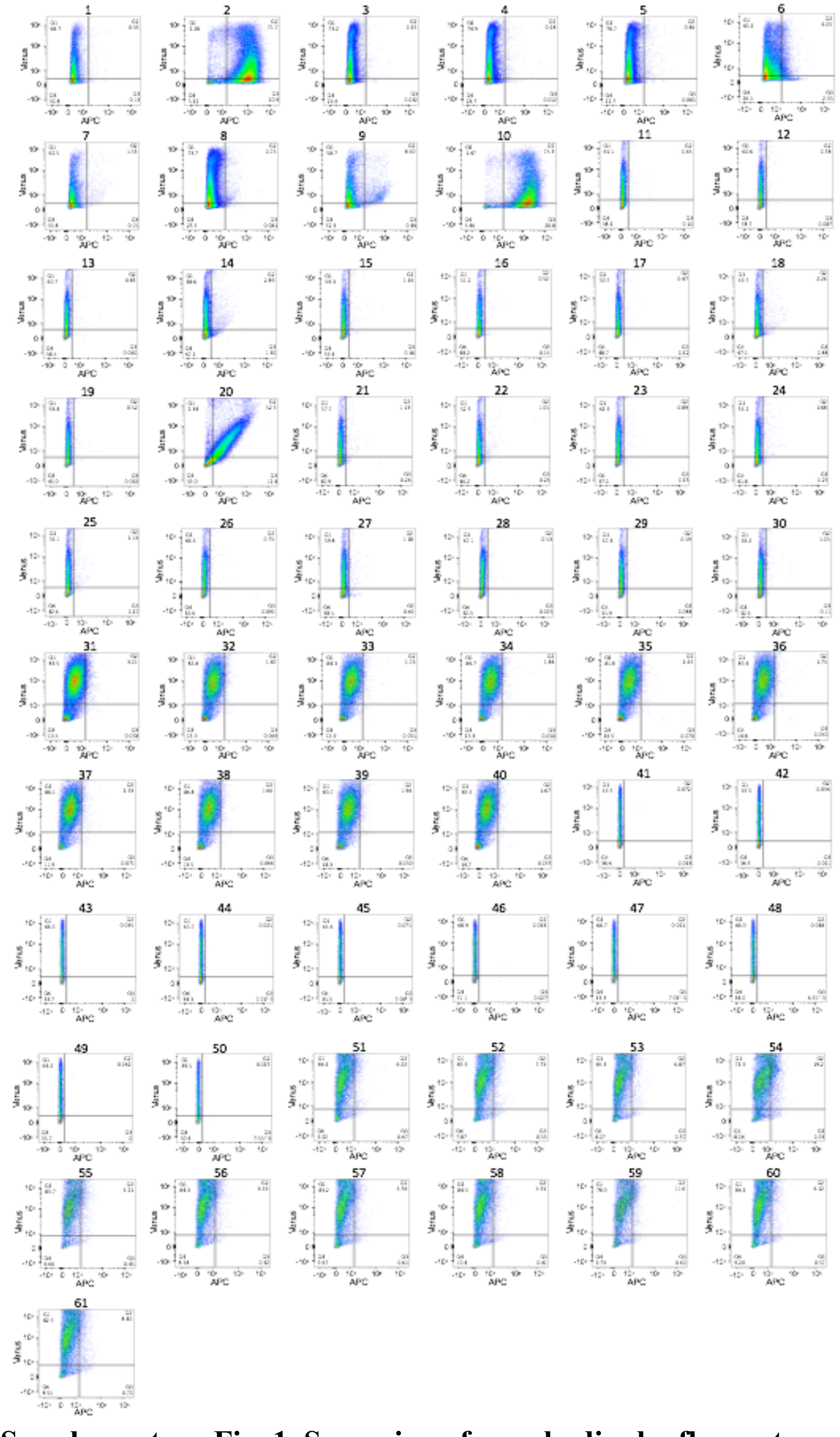

Supplementary Fig. 1. Screening of nanobodies by flow cytometry

Analysis of nanobody binding to mGlyR by flow cytometry in HEK293 cells transiently transfected with mGlyR and incubated with individual nanobody clones and anti-myc-APC conjugated antibody. mGlyR-Venus intensity is plotted on Y axis and X axis represents APC signal (nanobody binding). Percentages of cells in each quadrant are indicated. Good candidate shows specific binding (high value in Q2 quadrant and low Q3).

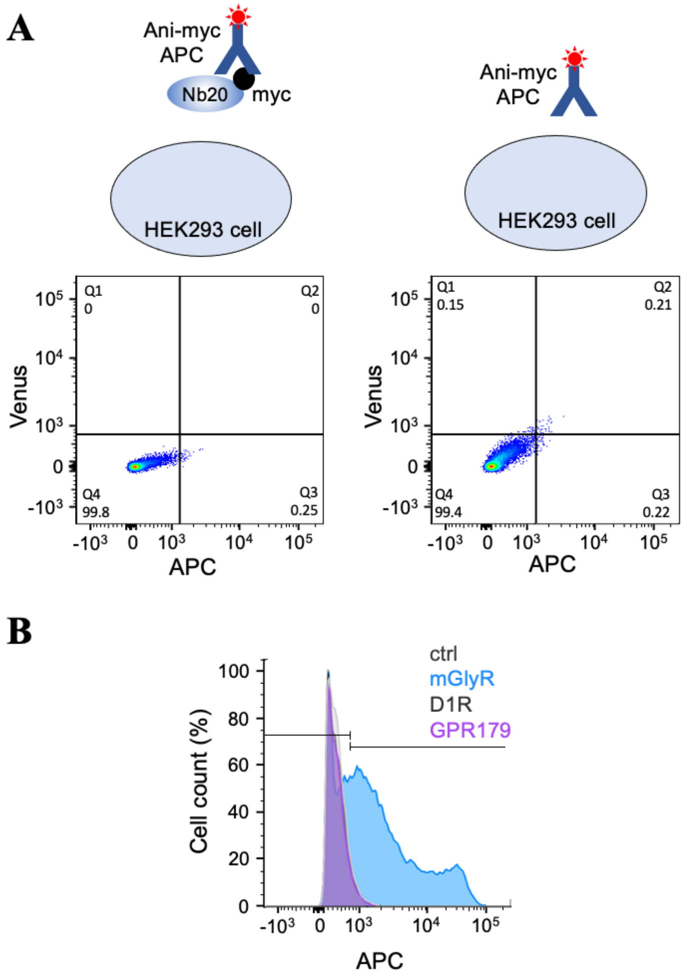

Supplementary Fig. 2. Specificity of Nb20 for mGlyR

A) Schematic of the detection strategy and analysis of the binding. Anti-myc APC conjugated antibody ± Nb20 were incubated on cells not expressing mGlyR. Percentages of cells in each quadrant are indicated. **B**) Analysis of nanobody binding by flow cytometry of HEK suspension cells transiently expressing mGlyR or other receptors incubated with Nb20 and anti-myc-APC conjugated antibody.

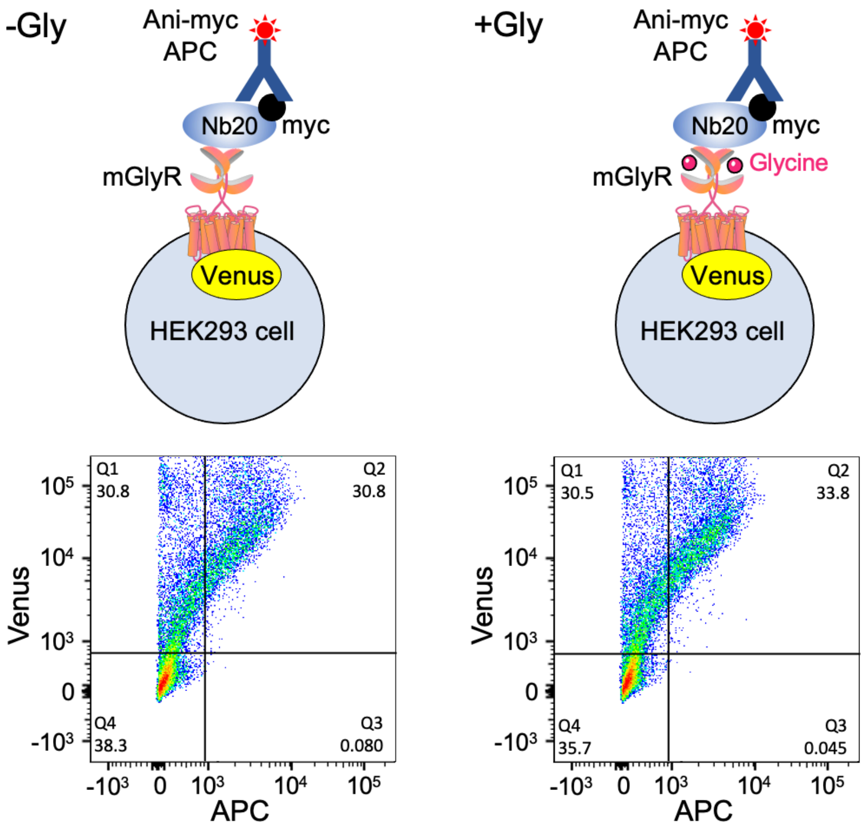

Supplementary Fig. 3. The effect of glycine on Nb20 binding to mGlyR
HEK293 cells transfected with mGlyR were incubated with Nb20 (1µM) and anti-myc APC conjugated antibody in the presence or absence of 100 µM of glycine.

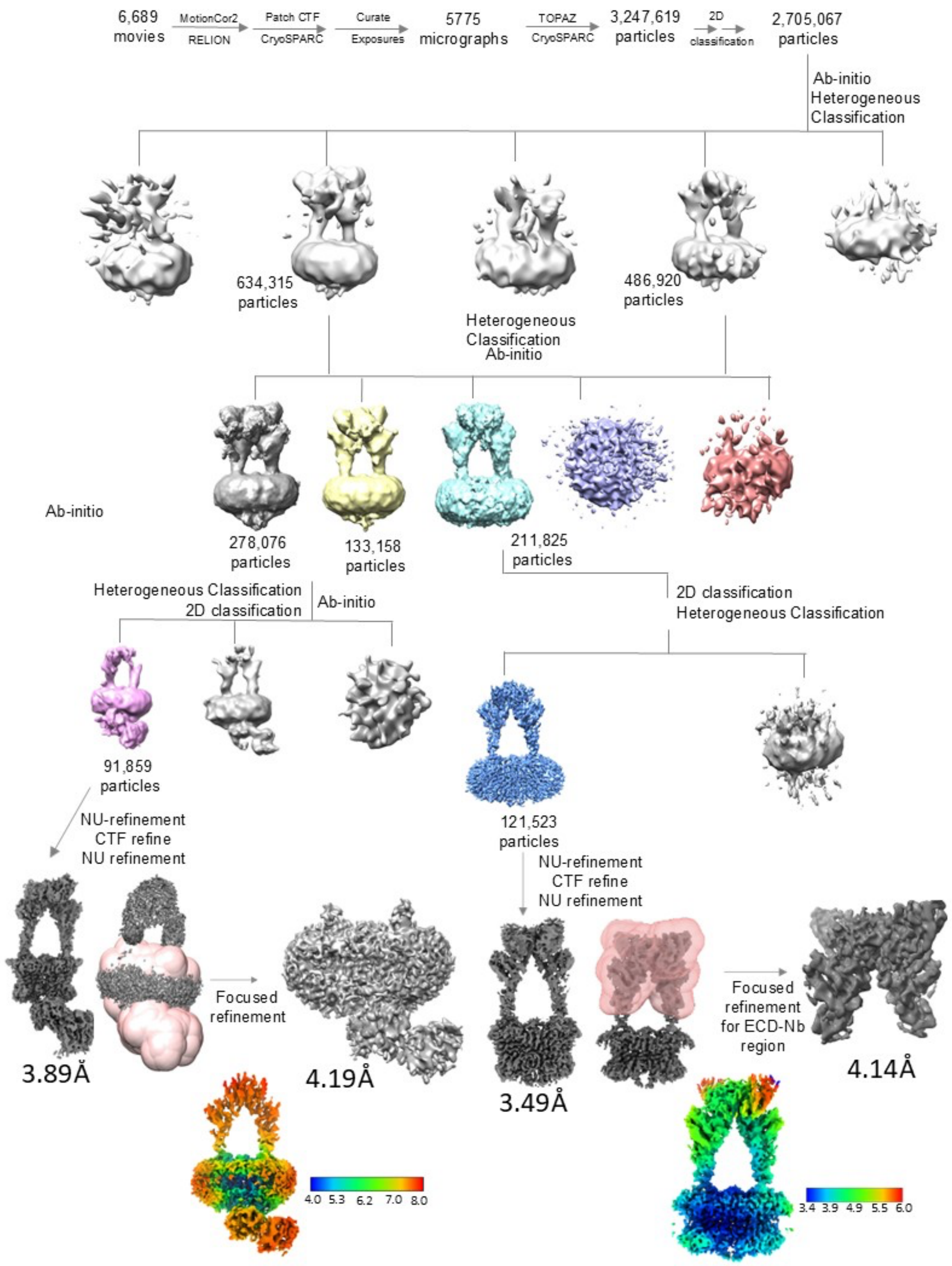

Supplementary Fig. 4. Workflow of cryo-EM data processing
Cryo-EM processing steps to obtain high resolution maps of human mGlyR-Nb20 and Nb20-mGlyR-RGS7-Gβ5 complexes.

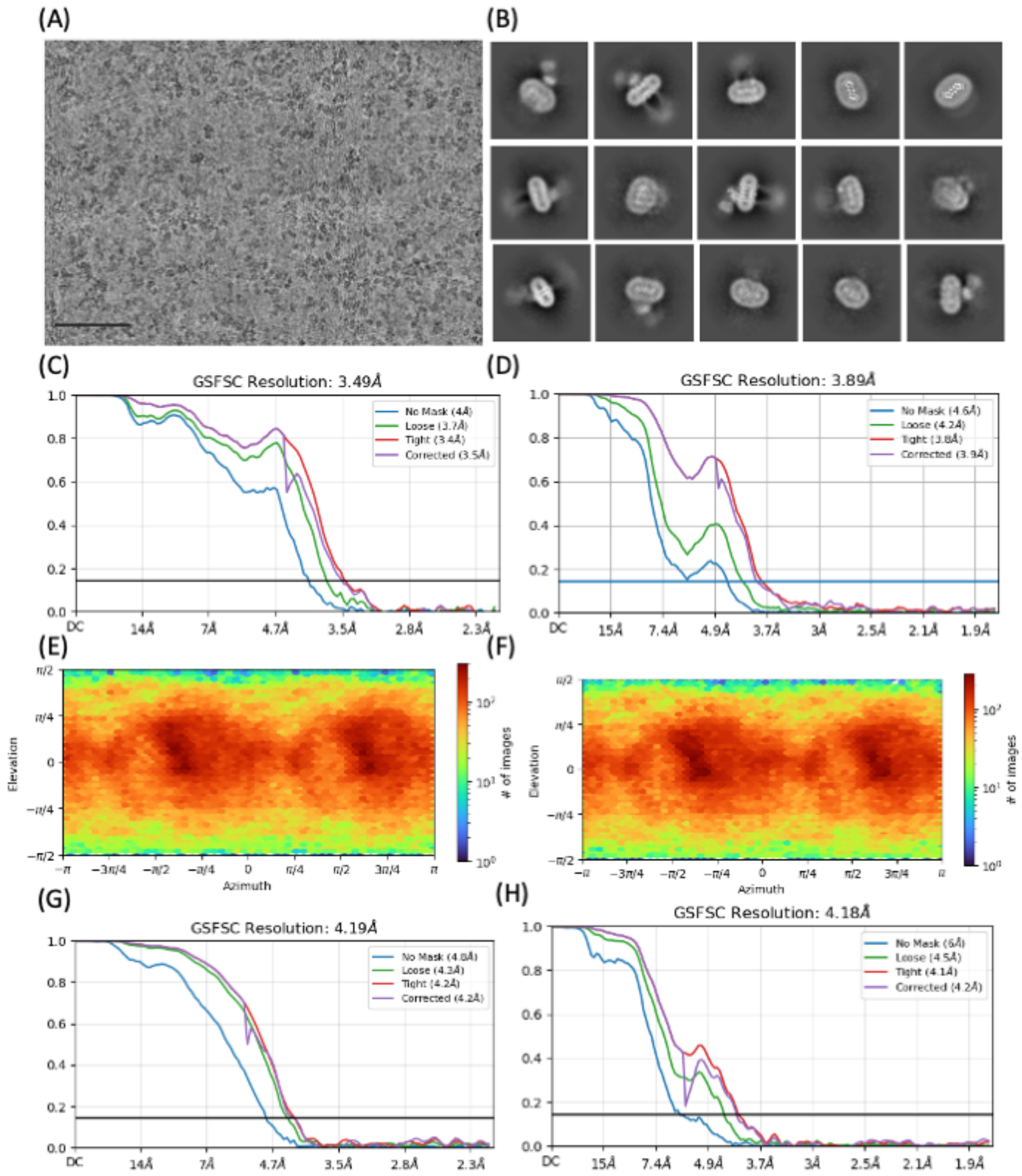

Supplementary Fig. 5. Cryo-EM data processing of human mGlyR-Nb20 and Nb20-mGlyR-RGS7-Gβ5 complexes

A) Representative cryo-EM micrograph, the scale bar corresponds to 100 nm. **B**) Representative 2D class averages for mGlyR-Nb20 and Nb20-mGlyR-RGS7-Gβ5 complex. **C**) and **D**) Gold standard Fourier shell correlation (FSC) curves (Resolutions reported for the maps determined by an FSC cut-off value of 0.143, shown in blue line) for mGlyR-Nb20 (3.49 Å) (D) Nb20-mGlyR-RGS7-Gβ5 complex (3.89 Å). **E**) and **F**) Euler angle orientation distribution plots, from CryoSPARC, for the final maps of mGlyR-Nb20 (D) and Nb20-mGlyR-RGS7-Gβ5 complexes. **G**) FSC curves for local refinements focused on the ECD–Nb20 (4.14 Å), **H**) FSC curves for local refinements focused on TM–RGS7/Gβ5 (4.18 Å).

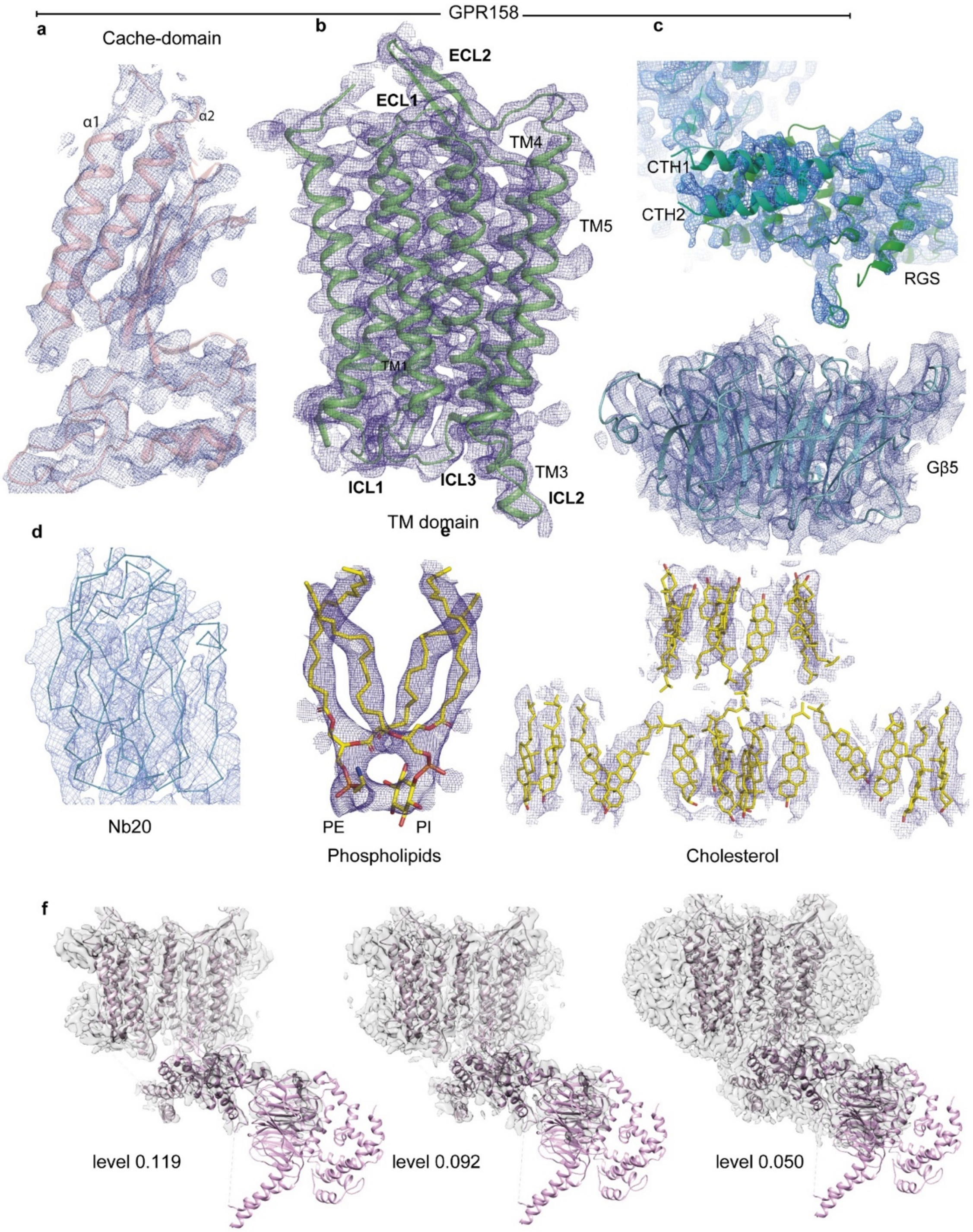

Supplementary Fig. 6. EM density map shown as mesh with corresponding models
A) ECD domain, **B**) TM domain, **C**) RGS and Gβ5, **D**) nanobody Nb20, **E**) phospholipids and cholesterols, **F**) focused refinement of the TM domain and RGS7/Gβ5 complex at different contour levels. The density corresponding to the RGS domain is not observed in the map, indicating its possible flexibility or absence in the focused refinement.

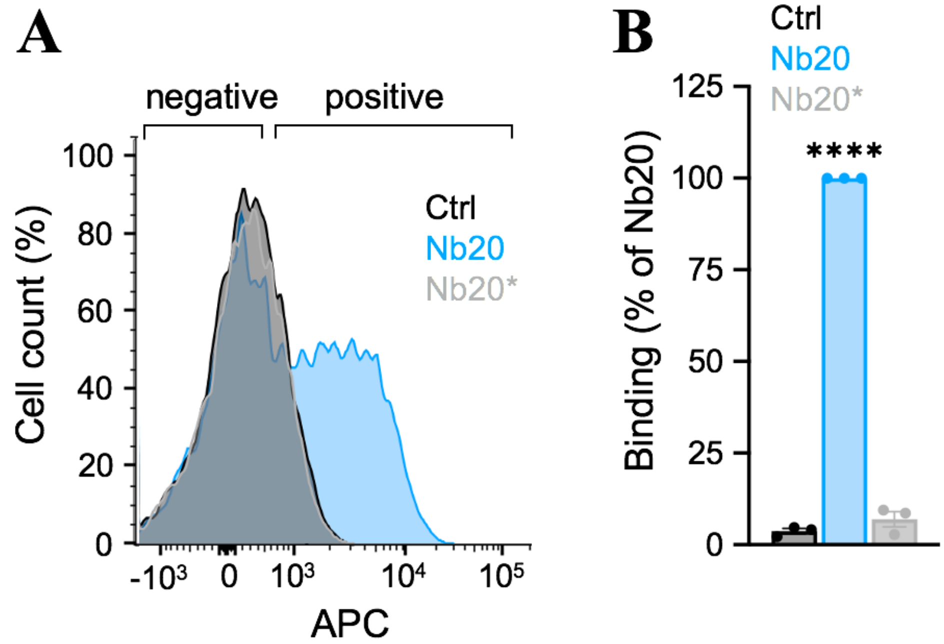

Supplementary Fig. 7. Validation of Nb20 binding determinants by mutagenesis
A) The binding ability of Nb20* on mGlyR was monitored by flow cytometry. 1.10^6 cells transiently transfected to express mGlyR were incubated with 1µM of Nb20 or Nb20* or vehicle and anti-myc APC conjugated antibody and binding was measured. **A**) representative histogram of the binding measurement. **B**) Quantification of anti-myc-APC binding detected in flow cytometry experiments. Data represent mean ± SEM of n = 3 independent experiments. ns = p=0.2264>0.05, ****$p < 0.0001$, one-way ANOVA.

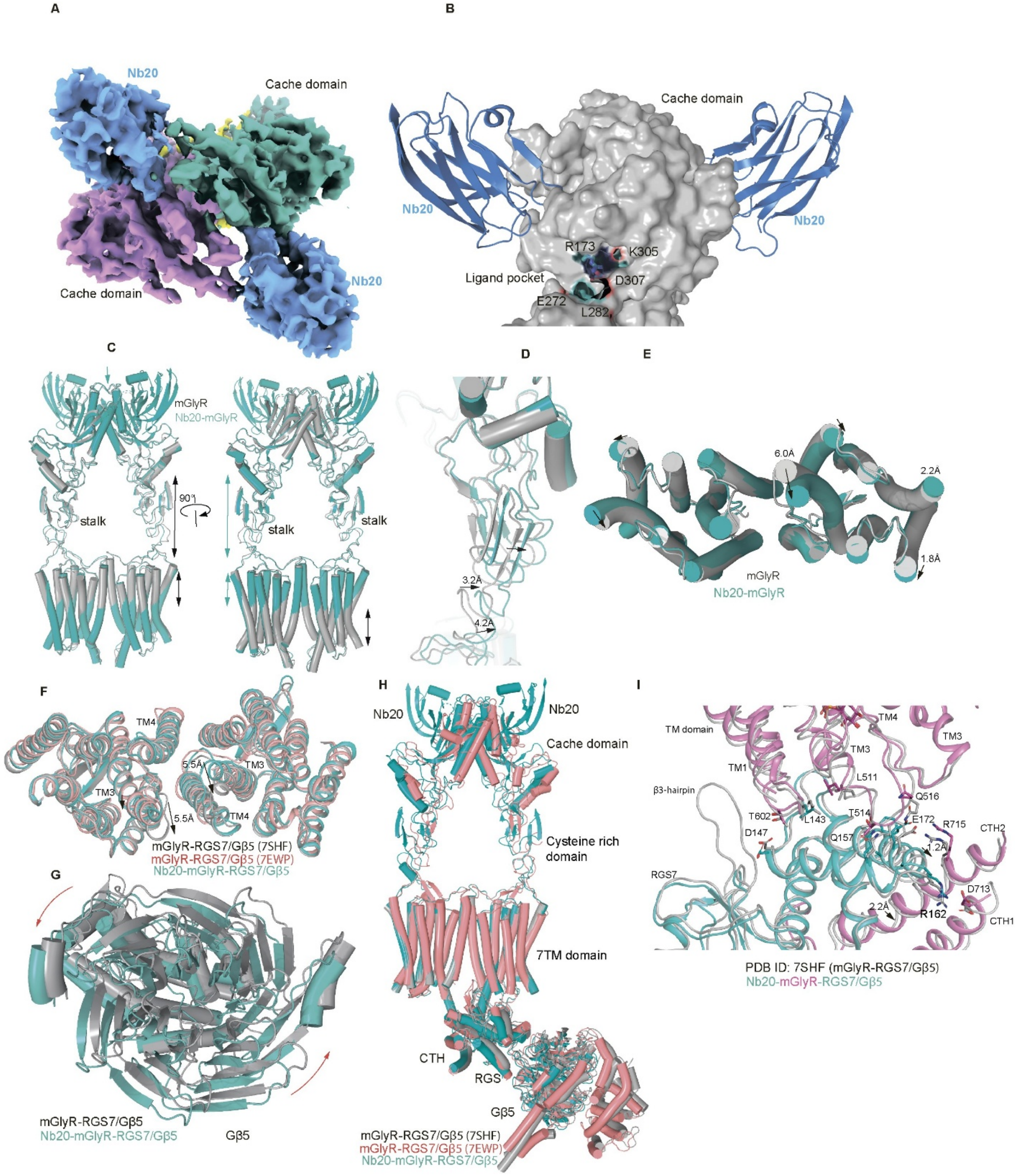

Supplementary Fig. 8. Cryo-EM structures of Nb20-bound mGlyR

A) Top view of the cryo-EM map showing Nb20 bound to mGlyR. **B**) Putative ligand binding pocket view highlighting residues involved in ligand binding. **C**) Side view and 90° rotated view of the global structural superimposition of mGlyR-Nb20 and mGlyR-apo illustrating relative domain movements. The two orthogonal views (90° apart) emphasize the inward and outward shifts of the extracellular and intracellular halves of the TM domain. **D**) Comparison of the stalk domain between mGlyR-Nb20 and mGlyR-apo structures with global alignment. **E**) Structural changes in

7TM region of mGlyR-apo compared to Nb20-mGlyR as viewed from the extracellular side. **F**) Structural changes in 7TM region of mGlyR-RGS7/Gβ5 (PDB: 7EWP, 7SHF) compared to Nb20-mGlyR-RGS7/Gβ5 as viewed from the cytoplasmic side. **H)** TM-anchored structural superimposition of mGlyR-RGS7/Gβ5 with Nb20-mGlyR-RGS7/Gβ5. **G**) Bottom view of Gβ5 from the alignment in panel H. **I**) Remodelling of interactions interface as revealed by structural superimposition of mGlyR–Nb20–RGS7/Gβ5 and mGlyR–RGS7/Gβ5 complexes (PDB: 7SHF, and 7EWP shown in grey and salmon respectively).

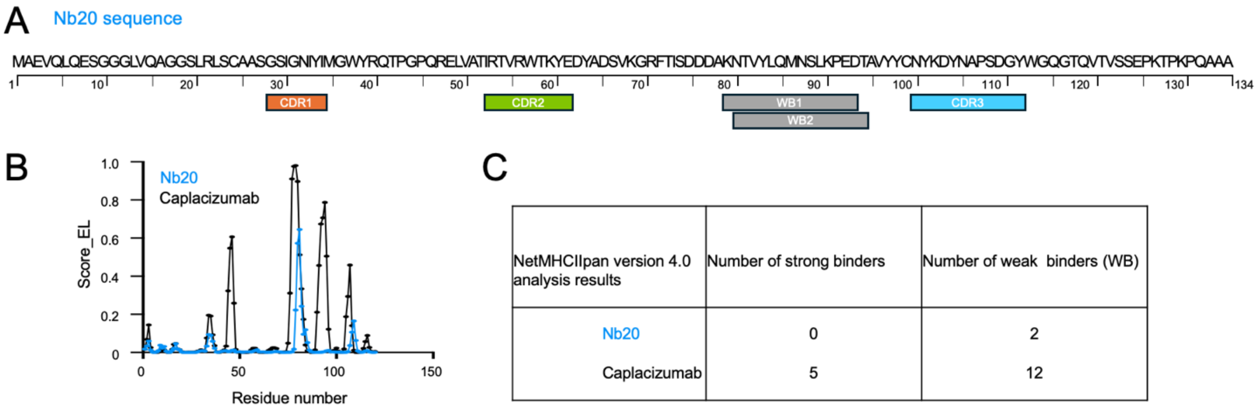

NetMHCIIpan version 4.0 analysis results	Number of strong binders	Number of weak binders (WB)
Nb20	0	2
Caplacizumab	5	12

Supplementary Fig. 9. Analysis of Nb20 immunogenicity
A) Sequence of Nb20 with indicated features that include position of the CDR regions and predicted class II MHC weak binding epitopes (WB). **B**) Distribution potential immunogenic regions across Nb20 sequence in comparison to FDA-approved nanobody Caplacizumab predicted by the NetMHCIIpan software. **C**) Summary of identified MHC class II binders in Nb20 and Caplacizumab.

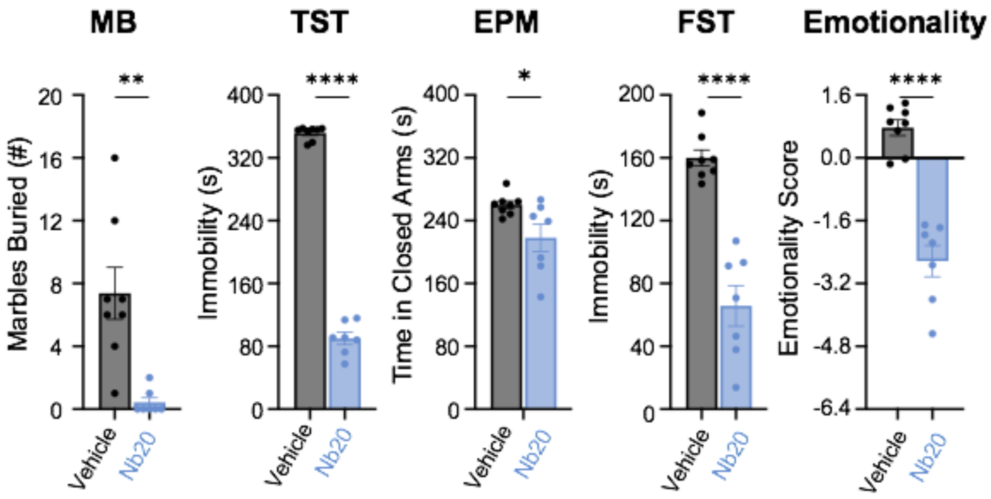

Supplementary Fig. 10. Anti-depressant effects of Nb20 persists for two weeks after administration in mice

Mice were evaluated in elevated plus maze (EPM), marble burying (MB), tail suspension test (TST), and forced swim test (TST) paradigms 2 weeks after ICV injection of Nb20 or vehicle control. (n = 8 mice in vehicle (4 males and 4 females) and 7 (4 males and 3 females) in Nb20 treated groups). Calculation of emotionality scores based on superscoring of four behavioral tests. Data are mean ± SEM. Unpaired t-test, MB $^{**}p = 0.0019<0.01$, TST $^{****}p<0.0001$, EPM $^{*}p = 0.0273<0.05$, FST $^{****}p<0.0001$, Emotionality $^{****}p<0.0001$.

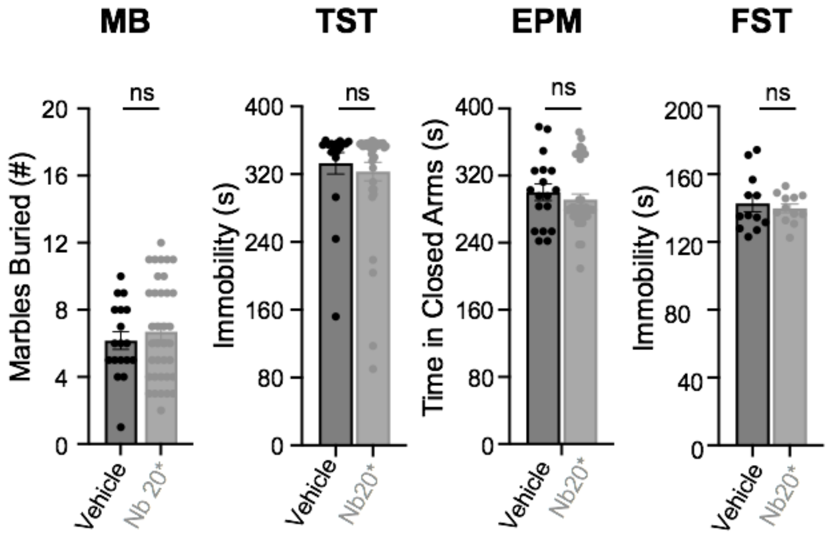

Supplementary Fig. 11. Effect of the control nanobody (Nb20*) in mice
Mutated Nb20* incapable of binding to mGlyR or vehicle were injected ICV in naïve mice. Mice Mutated Nb20* (9.6 μg) incapable of binding to mGlyR or vehicle were injected ICV in naïve mice. Mice were evaluated in elevated plus maze (EPM), marble burying (MB), tail suspension test (TST), and forced swim test (TST) paradigms (n = 18 mice in vehicle (9 males and 9 females) and 36 (18 males and 18 females) in Nb20* treated groups). Data are mean ± SEM. Unpaired t-test, MB p = 0.5096>0.05, TST p = 0.5859>0.05, EPM p = 0.4580>0.05, FST p = 0.6079>0.05.

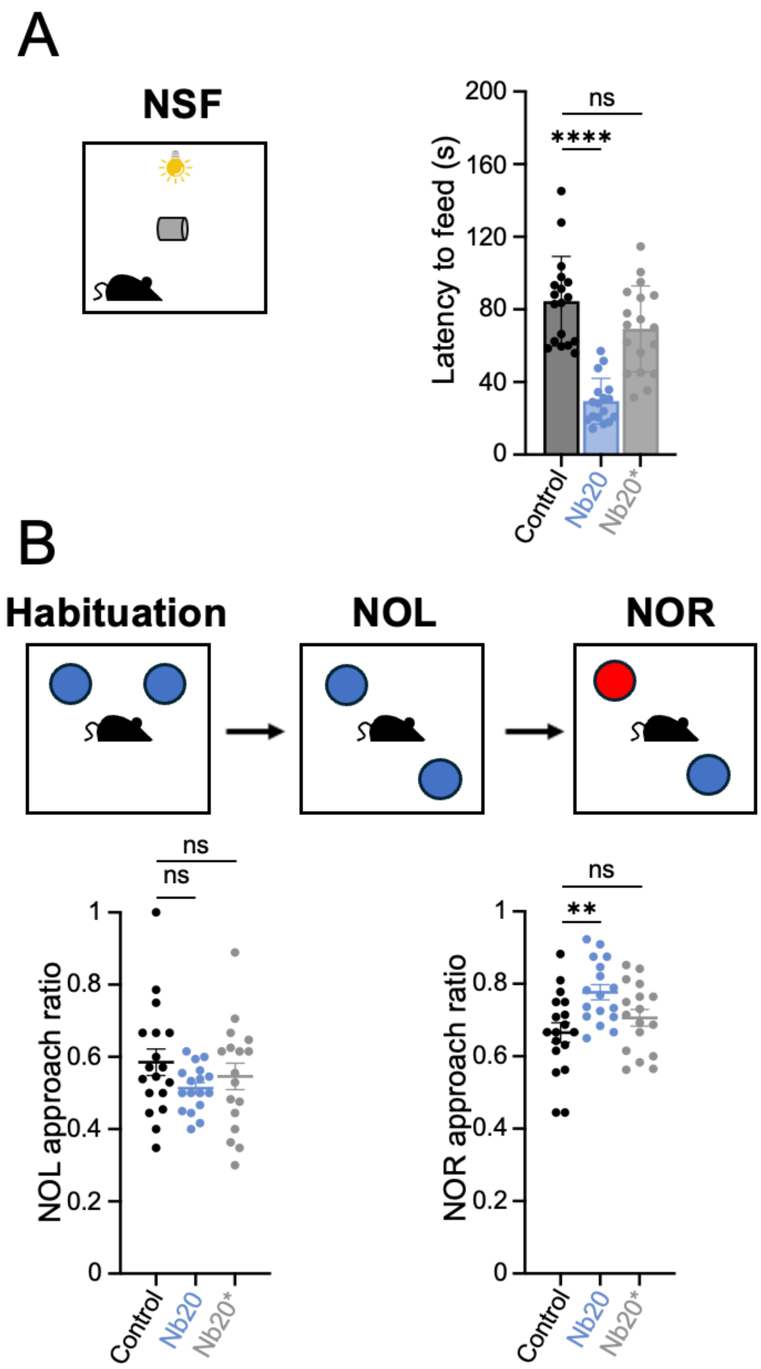

Supplementary Fig. 12. Effect of Nb20 on motivation to feed and cognition

A) Performance of mice treated with Nb20 or control inactive Nb20* in a novelty-suppressed feeding (NSF) task. Data are mean ± SEM. One-way ANOVA, Tukey's test. NSF: $F(2,50)=31.31$, $p<0.001$ Control vs Nb20 ***$p<0.0001$, Control vs Nb20*: $p = 0.0896>0.05$. **B**) Evaluation of mice treated with Nb20 or control inactive Nb20* by a novel object location (NOL) and novel object recognition (NOR) tests. Data are presented as mean ± SEM. (n= 17-18 mice per group). One-way ANOVA, Tukey's test. NOL: $F(2,49)=1.35$, $p=0.2668$. NOR: $F(2,49)=5.553$, $p=0.0067$, Control vs Nb20: **$p= 0.005< 0.01$, Control vs Nb20* $p = 0.4545>0.05$.

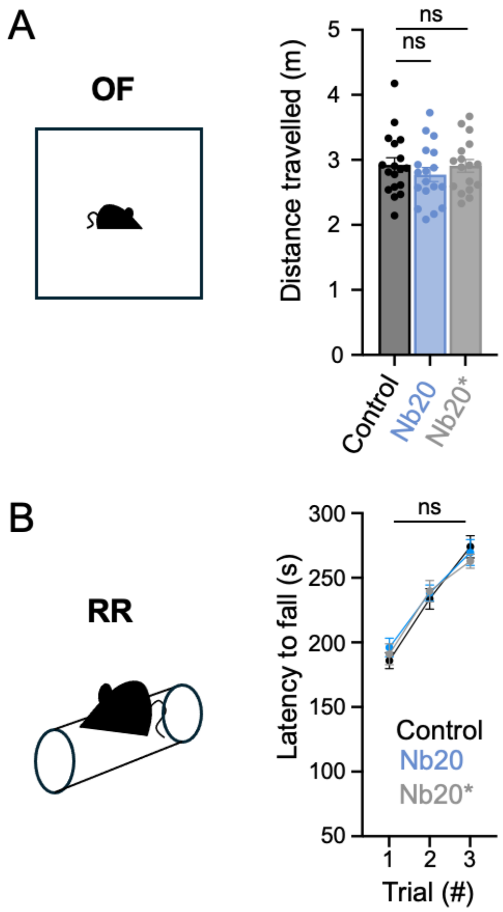

Supplementary Fig. 13. Effect of Nb20 on motor behavior of mice
A) Evaluation of locomotor activity in mice treated with Nb20 or control inactive Nb20* in an open field (OF) task. Data are mean ± SEM. One-way ANOVA $F(2,50)=0.6946$, $p=0.5556$. **B**) Testing motor performance of mice treated with Nb20 or control inactive Nb20* in an accelerating rotarod (RR). Data are presented as mean ± SEM. (n= 17-18 mice per group). ns, $p>0.05$. Two-way repeated measures ANOVA, treatment: $F(2,50)=0.1379$, $p=0.8715>0.05$, trial $F(1.8,91.1)=100.4$, $p<0.0001$, treatment x trial: $F(3.6, 91.1)=0.6098$, $p=0.6416>0.05$.

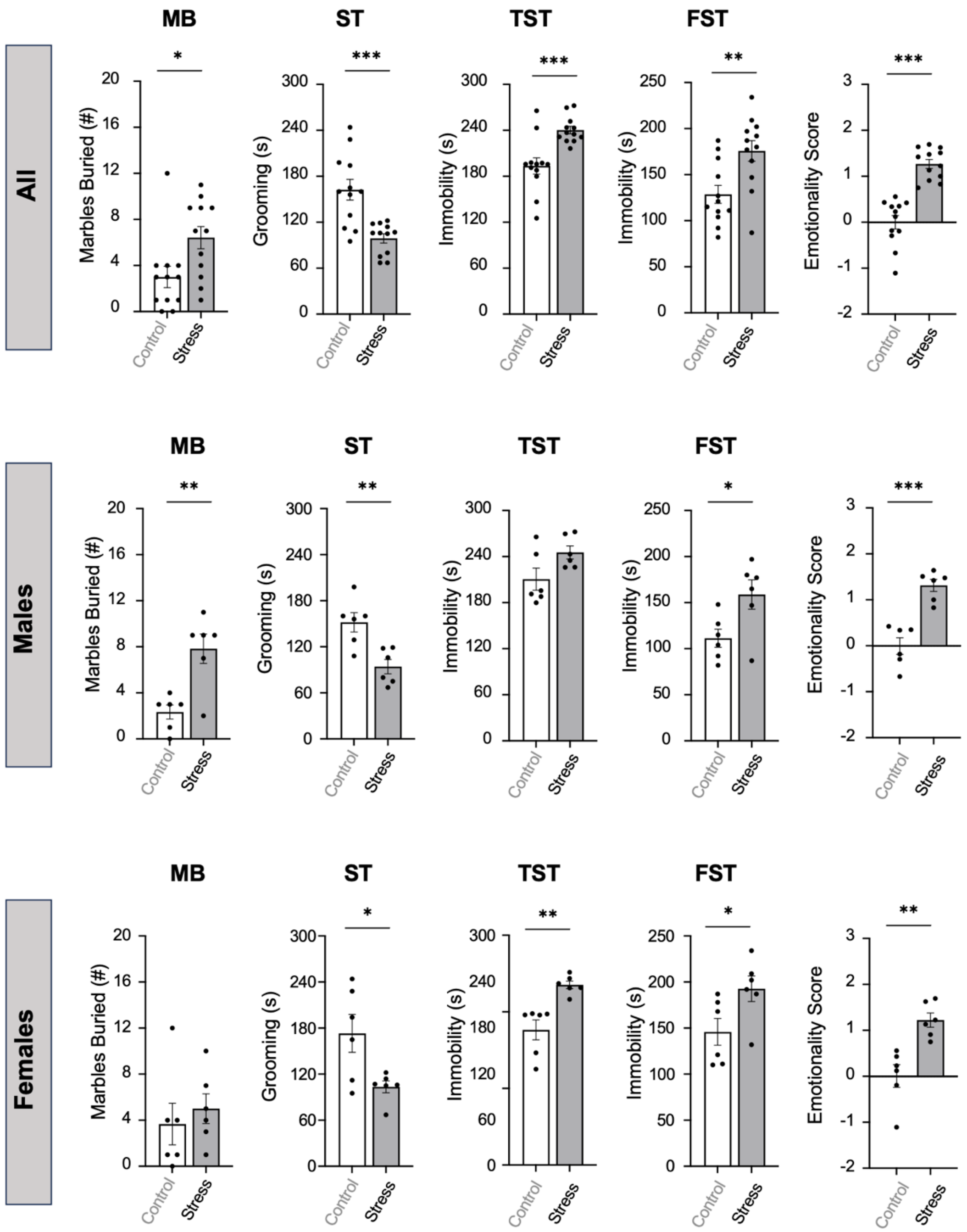

Supplementary Fig. 14. Evaluation of stress paradigm in vehicle injected mice

Evaluation of stressed vs non-stressed mice injected with vehicle control in a panel of behavioral tests consisting of marble burying (MB), splash test (ST), tail suspension test (TST), and forced swim test (TST) (n = 12 mice in both groups (6 males and 6 females). Calculation of emotionality scores based on superscoring of four behavioral tests. Data are mean ± SEM. Unpaired t-test, All : MB *p = 0.0182<0.05, ST ***p =0.0003<0.001, TST ***p =0.0006<0.001, FST **p = 0.004<0.01, Emotionality score ****p<0.0001 ; Males : MB **p = 0.003<0.01, ST **p =0.0042<0.01, TST p =0.0628>0.5, FST *p = 0.0292<0.05, Emotionality score ***p = 0.0002<0.001 ; Females : MB p = 0.5608>0.5, ST *p =0.0231<0.05, TST **p =0.0018<0.01, FST *p = 0.0420<0.05, Emotionality score **p = 0.0019<0.01.

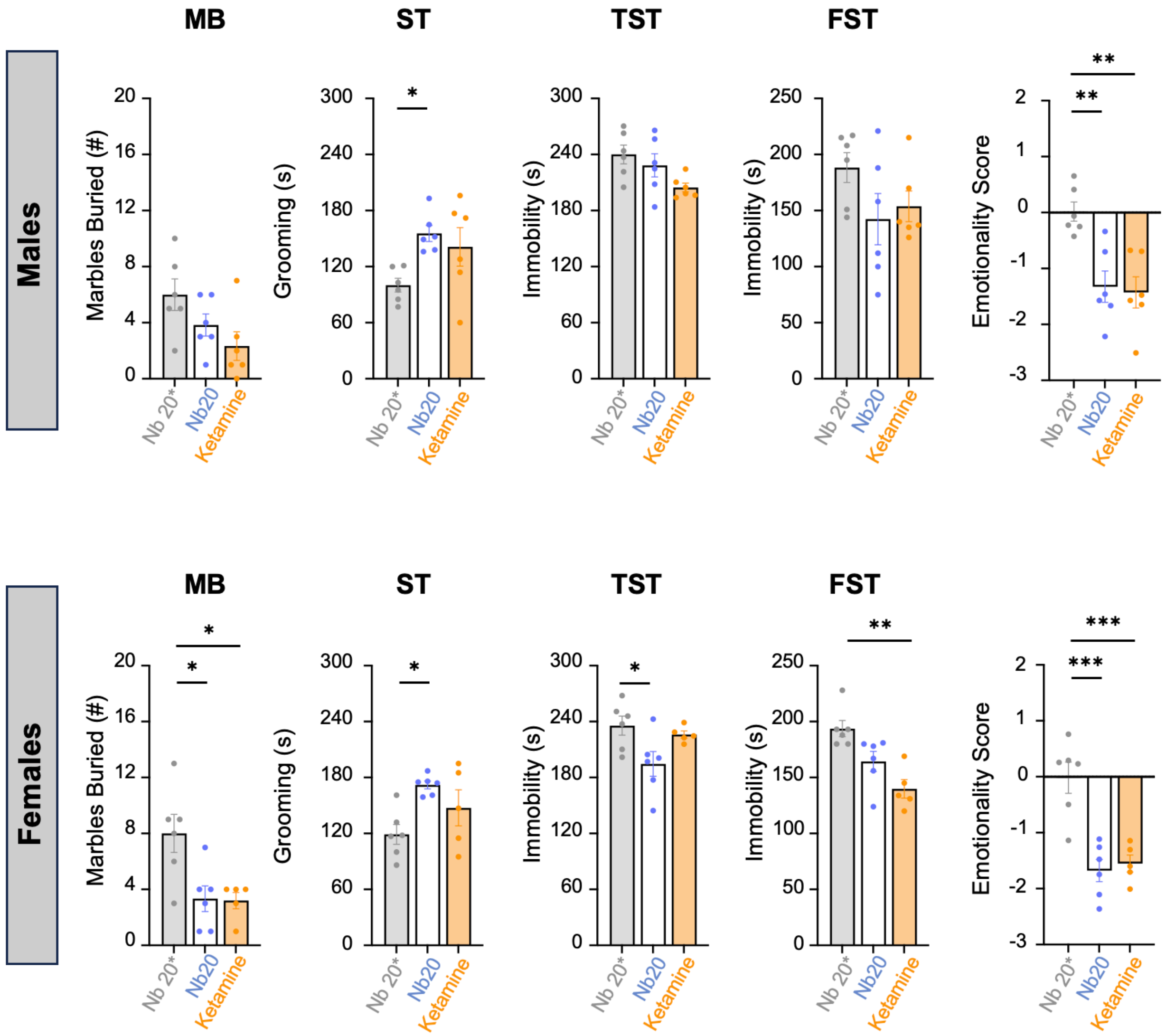

Supplementary Fig. 15. Sex dependence of mouse performance in stress-induced depression model

Following indicated treatments mice were evaluated using a panel of behavioral paradigms (n = 6 males and 5-6 females). Calculation of emotionality scores based on superscoring of four behavioral tests. Data are mean ± SEM. One-way ANOVA, Tukey's test Males : MB $F(2,15)=3.469$, $p=0.0578>0.05$, ST $F(2,15)=4.469$, $p=0.03$, Nb20*-Nb20 *p = 0.029<0.05, TST $F(2,15)=3.533$, $p=0.0553$, FST $F(2,15)=1.932$, $p=0.1792$, Emotionality score $F(2,15)=10.47$, $p=0.0014$, Nb20 **p=0.0046<0.01 Nb20* vs ketamine **p=0.0026<0.01 ; Females : MB $F(2,14)=6.895$, $p=0.0082$, Nb20*-Nb20 *p =0.0159<0.01 Nb20* vs ketamine *p = 0.0179<0.01, ST $F(2,14)=5.179$, $p=0.0207$, Nb20* vs Nb20 *p = 0.0161<0.05, TST $F(2,14)=4.403$, $p=0.0328$, Nb20* vs Nb20 *p = 0.0324<0.05, FST $F(2,14)=10.41$, $p=0.0017$, Nb20* vs ketamine **p = 0.0013<0.001, Emotionality score $F(2,14)=17.66$, $p=0.0001$, Nb20* vs Nb20 ***p=0.0003<0.001 Nb20* vs ketamine ***p=0.0008<0.001.

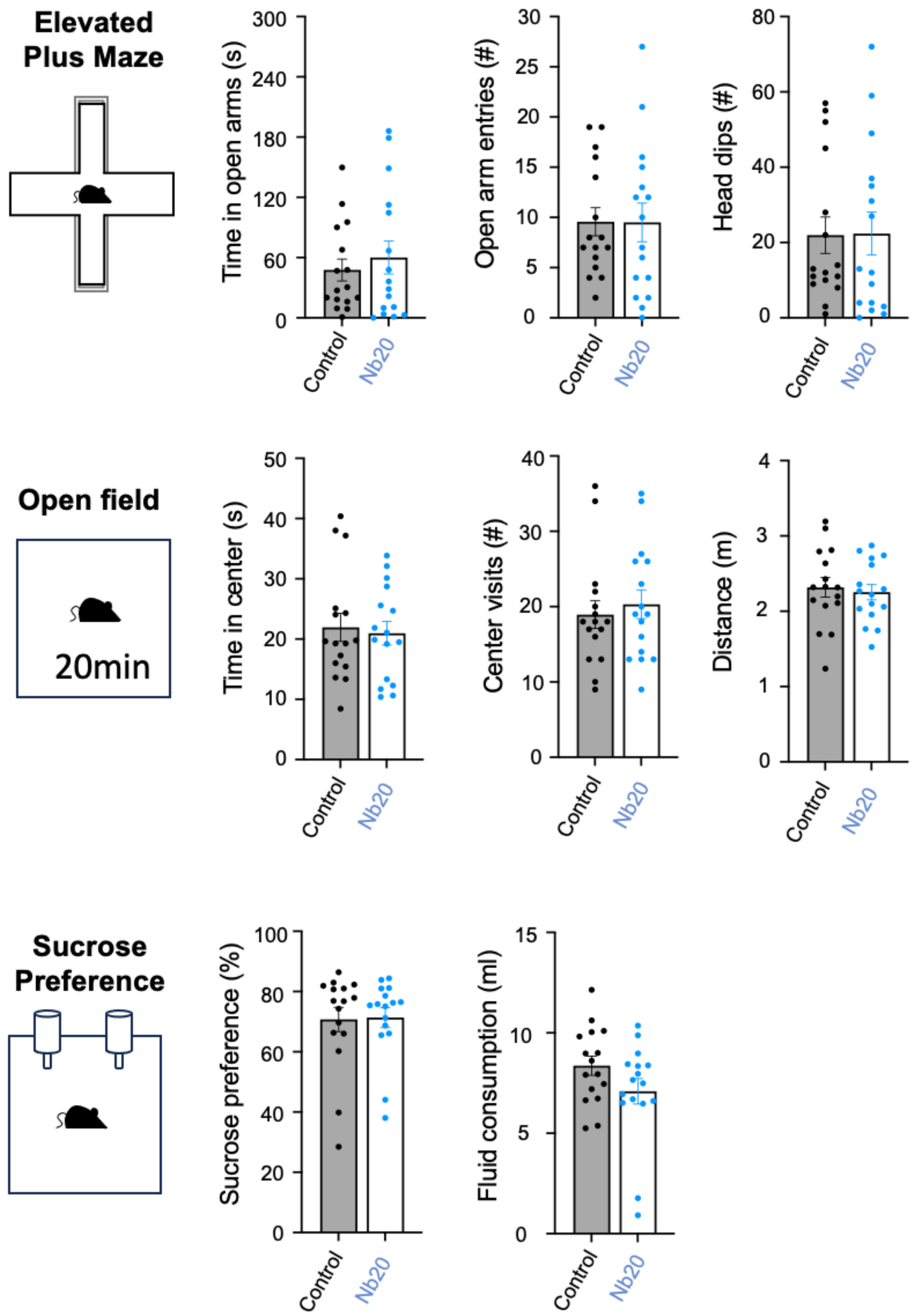

Supplementary Fig. 16. Evaluation of mouse behavior in a stress-induced depression model Stressed mice were treated with intranasal delivery of vehicle or Nb20 and their behavior was evaluated in the indicated paradigms. n = 16 mice (8 males and 8 females). Data are mean ± SEM. No statistically significant effects ($p>0.05$) were observed when evaluated by unpaired t-test.

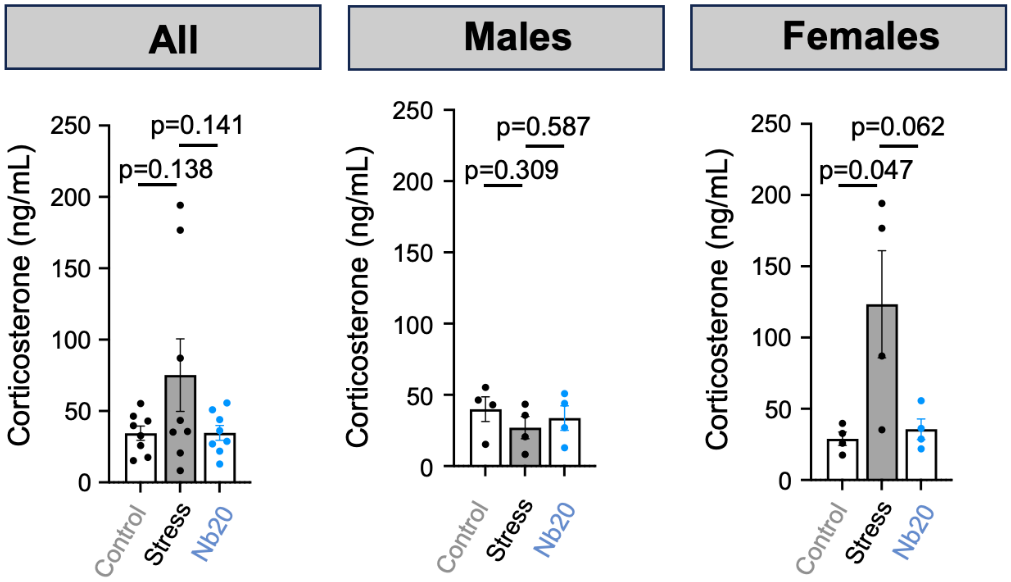

Supplementary Fig. 17. Effect of stress and Nb20 treatment on corticosterone levels
Mice were subjected to CVS stress paradigm, treated with Nb20 and blood was collected 24h following the treatment. Corticosterone levels were measured and plotted. Data are mean ± SEM. n=8 (4 males and 4 females), results of unpaired t-test are reported on the graphs. One-way ANOVA also reports significant differences in females, $F(2,9)=5.593$, $p=0.026<0.05$, with Tukey's post hoc values of 0.036 for Control vs Stress and 0.051 for Stress vs Nb20.

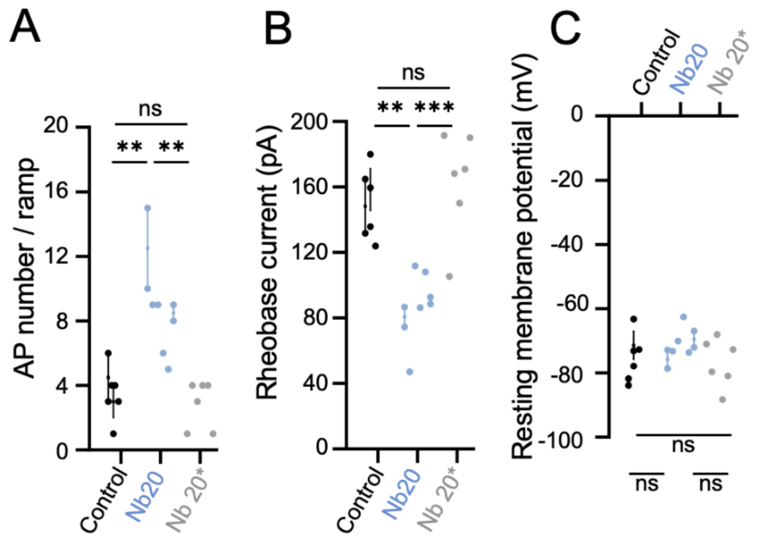

Supplementary Fig. 18. Effect of Nb20 on neuronal excitability evaluated by nested one-way ANOVA.

A) Quantification of changes in excitability by number of action potentials fired in response to 200 pA current ramp (n = 6-8 neurons, 3-6 mice). Nested One-way ANOVA; followed by Tukey test, **p<0.01. **B**) Quantification of changes in excitability by rheobase current (n = 6-8 neurons, 3-6 mice). Nested One-way ANOVA; followed by Tukey test, ***p<0.001, **p<0.01. **C**) Resting membrane potential of layer II-III pyramidal neurons in WT mice (n = 6-8 neurons, 3-6 mice). Nested One-way ANOVA; followed by Tukey test, ns=p>0.05.
