# Supplementary material for: Targeting mGlyR with nanobodies for depression
Source: Nat Commun. 2026 Jan 26;17:831. doi: 10.1038/s41467-026-68339-x (PMC12834959; doi:10.1038/s41467-026-68339-x)
Supplement: Supplementary file 2 — Reporting Summary [file 41467_2026_68339_MOESM2_ESM.pdf]

nature portfolio

Corresponding author(s): Dr. Kirill Martemyanov

Last updated by author(s): Dec 2, 2025

Reporting Summary

Nature Portfolio wishes to improve the reproducibility of the work that we publish. This form provides structure for consistency and transparency in reporting. For further information on Nature Portfolio policies, see our Editorial Policies and the Editorial Policy Checklist.

Statistics

For all statistical analyses, confirm that the following items are present in the figure legend, table legend, main text, or Methods section.

n/a	Confirmed	
☐	☒	The exact sample size (n) for each experimental group/condition, given as a discrete number and unit of measurement
☐	☒	A statement on whether measurements were taken from distinct samples or whether the same sample was measured repeatedly
☐	☒	The statistical test(s) used AND whether they are one- or two-sided<br>*Only common tests should be described solely by name; describe more complex techniques in the Methods section.*
☐	☒	A description of all covariates tested
☐	☒	A description of any assumptions or corrections, such as tests of normality and adjustment for multiple comparisons
☐	☒	A full description of the statistical parameters including central tendency (e.g. means) or other basic estimates (e.g. regression coefficient) AND variation (e.g. standard deviation) or associated estimates of uncertainty (e.g. confidence intervals)
☒	☐	For null hypothesis testing, the test statistic (e.g. F, t, r) with confidence intervals, effect sizes, degrees of freedom and P value noted<br>*Give P values as exact values whenever suitable.*
☒	☐	For Bayesian analysis, information on the choice of priors and Markov chain Monte Carlo settings
☒	☐	For hierarchical and complex designs, identification of the appropriate level for tests and full reporting of outcomes
☒	☐	Estimates of effect sizes (e.g. Cohen's d, Pearson's r), indicating how they were calculated

Our web collection on statistics for biologists contains articles on many of the points above.

Software and code

Policy information about availability of computer code

Data collection	n/a
Data analysis	n/a

For manuscripts utilizing custom algorithms or software that are central to the research but not yet described in published literature, software must be made available to editors and reviewers. We strongly encourage code deposition in a community repository (e.g. GitHub). See the Nature Portfolio guidelines for submitting code & software for further information.

Data

Policy information about availability of data

All manuscripts must include a data availability statement. This statement should provide the following information, where applicable:

- Accession codes, unique identifiers, or web links for publicly available datasets
- A description of any restrictions on data availability
- For clinical datasets or third party data, please ensure that the statement adheres to our policy

The atomic coordinates have been deposited in the Protein Data Bank (PDB) under accession codes 9VOR: https://doi.org/10.2210/pdb9VOS/pdb and 9VOS: https://doi.org/10.2210/pdb9VOR/pdb.
Previously published atomic coordinates used in this study are 7SHF: https://doi.org/10.2210/pdb7SHF/pdb and 7EWP: https://doi.org/10.2210/pdb7EWP/pdb

The source data underlying Figure1, Figure 2, Figure 4, Figure 5 and Supplementary Figure S7 and Supplementary Figures 9-18 are provided as a Source Data file. The data generated in this study have been deposited in the Figshare database at the following link: https://doi.org/10.6084/m9.figshare.30714752.

Research involving human participants, their data, or biological material

Policy information about studies with human participants or human data. See also policy information about sex, gender (identity/presentation), and sexual orientation and race, ethnicity and racism.

Reporting on sex and gender	n/a
Reporting on race, ethnicity, or other socially relevant groupings	n/a
Population characteristics	n/a
Recruitment	n/a
Ethics oversight	n/a

Note that full information on the approval of the study protocol must also be provided in the manuscript.

Field-specific reporting

Please select the one below that is the best fit for your research. If you are not sure, read the appropriate sections before making your selection.

- [x] Life sciences
- [ ] Behavioural & social sciences
- [ ] Ecological, evolutionary & environmental sciences

For a reference copy of the document with all sections, see nature.com/documents/nr-reporting-summary-flat.pdf

Life sciences study design

All studies must disclose on these points even when the disclosure is negative.

Sample size	No statistical method was used to predetermine sample size for animal studies. The functional data shown represent at least 3 individual experiments each performed in triplicate.
Data exclusions	No data were excluded from the analyses.
Replication	The functional data shown represent at least 3 individual experiments each performed in triplicate.
Randomization	Animals were randomly assigned to experimental groups depending on genotype.
Blinding	Animal experimenters were blinded to the treatment groups.

Reporting for specific materials, systems and methods

We require information from authors about some types of materials, experimental systems and methods used in many studies. Here, indicate whether each material, system or method listed is relevant to your study. If you are not sure if a list item applies to your research, read the appropriate section before selecting a response.

Materials & experimental systems

n/a	Involved in the study	
[ ]	[x]	Antibodies
[ ]	[x]	Eukaryotic cell lines
[x]	[ ]	Palaeontology and archaeology
[ ]	[x]	Animals and other organisms
[x]	[ ]	Clinical data
[x]	[ ]	Dual use research of concern
[x]	[ ]	Plants

Methods

n/a	Involved in the study	
[x]	[ ]	ChIP-seq
[ ]	[x]	Flow cytometry
[x]	[ ]	MRI-based neuroimaging

Antibodies

Antibodies used	anti-myc-APC conjugated antibody (R&d Systems #IC3696A)<br>mouse anti-human IgG CH2 monoclonal antibody (Cytiva)

Validation	Anti-myc APC conjugated antibody was validated using flow cytometry control cells (without APC tag).<br>Mouse anti-human IgG CH2 antibody was validated using SPR negative control

Eukaryotic cell lines

Policy information about cell lines and Sex and Gender in Research

Cell line source(s)	HEK293FT cells were obtained from ThermoFisher (R70007)
Authentication	cells were sold with authentication certificate
Mycoplasma contamination	cells were frequently tested for mycoplasma contamination. Never tested positive
Commonly misidentified lines (See ICLAC register)	*Name any commonly misidentified cell lines used in the study and provide a rationale for their use.*

Animals and other research organisms

Policy information about studies involving animals; ARRIVE guidelines recommended for reporting animal research, and Sex and Gender in Research

Laboratory animals	mouse (Mus musculu) strain C57/Bl6 were used at 8 weeks old
Wild animals	n/a
Reporting on sex	both gender were used with equal distribution in each group
Field-collected samples	n/a
Ethics oversight	All animal experiments were approved by UF Scripps Biomedical Research Institutional Animal Care and Use Committee (IACUC), protocol 16-032 and performed in accordance with NIH guidelines.

Note that full information on the approval of the study protocol must also be provided in the manuscript.

Plants

Seed stocks	n/a
Novel plant genotypes	n/a
Authentication	n/a

Flow Cytometry

Plots

Confirm that:

- [x] The axis labels state the marker and fluorochrome used (e.g. CD4-FITC).
- [x] The axis scales are clearly visible. Include numbers along axes only for bottom left plot of group (a 'group' is an analysis of identical markers).
- [x] All plots are contour plots with outliers or pseudocolor plots.
- [x] A numerical value for number of cells or percentage (with statistics) is provided.

Methodology

Sample preparation	HEK293FT cells were cultured in 6 well plates at the density of 1.106 per well and transfected with 2 µg of cDNA of mGlyR or empty pcDNA3.1+ in control experiments, using Metafectene Pro. 48h after transfection, cells were mechanically detached pipetting up-down, washed in PBS supplemented with 0.1% BSA, counted and incubated in PBS-0.1% BSA for 1h at 4°C under rotation. Nanobody-20 (Nb20) and 10 µl of anti-myc-APC conjugated antibody (R&d Systems #IC3696A) were added and incubated in the dark with rotation, at 4°C for 1h and washed 3 times before analysis in flow cytometer
Instrument	LSR-II BD flow cytometer

Software	FACS DIva was used to pilot the flow cytometer and FlowJo was used to analyse data
Cell population abundance	Cell population abundance was 10,000 cells in the final gate
Gating strategy	FSC-A/SSC-A first gate was used to discriminate cells to debris. Single cells were then sorted using FSC-A/FSC-H gating strategy. Finally, singlet mock transfected cells incubated with both Nanobody-myc tagged and anti-myc APC conjugated antibody were used to set negative threshold of venus (y-axis) and negative threshold of APC (x-axis).

☒ Tick this box to confirm that a figure exemplifying the gating strategy is provided in the Supplementary Information.
